# Reversible and permanent effects of tobacco smoke exposure on airway epithelial gene expression

**DOI:** 10.1186/gb-2007-8-9-r201

**Published:** 2007-09-25

**Authors:** Jennifer Beane, Paola Sebastiani, Gang Liu, Jerome S Brody, Marc E Lenburg, Avrum Spira

**Affiliations:** 1 Bioinformatics Program, Boston University, Cummington Street, Boston, MA 02215, USA; 2 The Pulmonary Center, Boston University Medical Center, Albany Street, Boston, MA 02118, USA; 3 School of Public Health, Boston University, Albany Street, Boston, MA 02118, USA; 4 Department of Genetics and Genomics, Boston University, Albany Street, Boston, MA 02118, USA

## Abstract

**Background:**

Tobacco use remains the leading preventable cause of death in the US. The risk of dying from smoking-related diseases remains elevated for former smokers years after quitting. The identification of irreversible effects of tobacco smoke on airway gene expression may provide insights into the causes of this elevated risk.

**Results:**

Using oligonucleotide microarrays, we measured gene expression in large airway epithelial cells obtained via bronchoscopy from never, current, and former smokers (*n *= 104). Linear models identified 175 genes differentially expressed between current and never smokers, and classified these as irreversible (*n *= 28), slowly reversible (*n *= 6), or rapidly reversible (*n *= 139) based on their expression in former smokers. A greater percentage of irreversible and slowly reversible genes were down-regulated by smoking, suggesting possible mechanisms for persistent changes, such as allelic loss at 16q13. Similarities with airway epithelium gene expression changes caused by other environmental exposures suggest that common mechanisms are involved in the response to tobacco smoke. Finally, using irreversible genes, we built a biomarker of ever exposure to tobacco smoke capable of classifying an independent set of former and current smokers with 81% and 100% accuracy, respectively.

**Conclusion:**

We have categorized smoking-related changes in airway gene expression by their degree of reversibility upon smoking cessation. Our findings provide insights into the mechanisms leading to reversible and persistent effects of tobacco smoke that may explain former smokers increased risk for developing tobacco-induced lung disease and provide novel targets for chemoprophylaxis. Airway gene expression may also serve as a sensitive biomarker to identify individuals with past exposure to tobacco smoke.

## Abstract 2

Oligonucleotide microarray analysis revealed 175 genes that are differentially expressed in large airway epithelial cells of people who currently smoke compared with those who never smoked, with 28 classified as irreversible, 6 as slowly reversible, and 139 as rapidly reversible.

## Background

Tobacco use remains the leading preventable cause of death in the United States, and cigarette smoking is the primary cause of chronic obstructive pulmonary disease and respiratory-tract cancers. Smoking is responsible for approximately 440,000 deaths per year in the US, resulting in 5.6 million years of potential life lost, $75 billion in direct medical costs, and $82 billion in lost productivity [1]. Exposure to tobacco smoke is widespread - approximately 45 million Americans are current smokers and 46 million are former smokers [2]. The risk of dying from smoking related diseases such as lung cancer and chronic obstructive pulmonary disease remains elevated for former smokers compared to never smokers [3]. In the Dorn Study of US veterans, the Kaiser Permanente Prospective Mortality Study, and American Cancer Society Cancer Prevention Study I (CPS-I) populations, the risk of death from lung cancer among former smokers was elevated above never smokers 20 or more years following cessation [4]. The Iowa Women's Health Study also found that former smokers had an elevated lung cancer risk compared with never smokers and that the risk for adenocarcinoma was elevated up to 30 years after quitting [5]. As an increasing fraction of current smokers become former smokers, more lung cancer cases will occur in former smokers as the absolute risk of lung cancer in the population declines [6]. It would be useful, therefore, to understand why former smokers remain at risk for lung cancer after smoking cessation in order to develop chemoprophylaxis treatments that might reduce risk.

A number of studies have shown that histologically normal large airway epithelial cells of current and former smokers with and without lung cancer display allelic loss [7,8], genomic instability [9], *p53 *mutations [10], changes in DNA methylation in the promoter regions of several genes (including *RARβ*, *H-cadherin*, *APC*, *p16*^*INK*4*a*^, and *RASFF1 *[11,12]), as well as changes in telomerase activity [13,14]. Many of the changes persist in smokers for years after cessation [8,9]. These observations suggest that the entire respiratory tree is affected by cigarette smoke, and that large airway cells might provide insight into the types and degree of epithelial cell injury that have occurred in current or former smokers.

We have previously reported a genome-wide expression profiling study of large bronchial airway epithelial cells obtained via bronchoscopy from never, current, and former smokers [15]. In that study, we defined the baseline airway gene expression profile among healthy never smokers and identified gene expression changes that occur in response to smoke exposure. Of note, we found that a subset of genes modulated by smoking did not return to baseline years after smoking cessation. However, the limited sample size of the former smoker group (*n *= 18) precluded a detailed study of gene expression reversibility post-smoking cessation.

In this study, we collected airway epithelial cells from a larger sample of never, current, and former smokers and developed statistical models to identify the gene expression changes associated with smoking and categorized the degree to which these are reversible upon smoking cessation. We further explored the relationship between these gene expression changes and a number of publicly available human bronchial epithelial microarray datasets. The comparison of our dataset with the other datasets provides insights into common mechanisms airway epithelial cells use in response to a variety of different toxins. Lastly, development of a biomarker for ever tobacco smoke exposure using genes irreversibly altered by cigarette smoke provided additional validation of the gene expression changes upon smoking cessation and may provide a useful tool for epidemiological studies.

## Results

### Patient population

Demographic information for the 21 never, 31 former, and 52 current smokers used in the present study are shown in Table 1. There were significant differences in age among the three groups (*P *< 0.05 by pairwise *t*-tests); however, there was no significant difference between cumulative tobacco exposure between the former and current smokers.

**Table 1:** Demographic information for the never, former, and current smokers

	Never	Former	Current
n	21	31	52
Age	32.3 (10.7)	55.9 (14.7)	48.6 (15.2)
Pack years		34.0 (30.1)	34.5 (34.2)
Months since quitting		145.2 (162.82)	

The mean and standard deviation (in parentheses) are reported. There is a significant age difference between the groups (*P *< 0.05 for all two-way group comparisons by *t*-test).

### Effect of smoking and smoking cessation

Three-hundred and forty-three probesets show significant differences in intensity between current and never smokers based on the significance of the current smoking status variable in the linear model (q-value < 0.05 corresponding to a *P *< 7.6 × 10^-4^; see Materials and methods). Two-hundred and nineteen probesets remained after applying a filter to retain only probesets where the absolute current smoking status coefficient was greater than or equal to 0.584 (corresponds to an age-adjusted fold change between current and never smokers of 1.5). Finally, after filtering out redundant probesets (probesets representing the same gene) from this set of 219 probesets, probesets representing 175 genes remained. There was a high degree of overlap (78%) between genes we previously identified as being perturbed by active cigarette smoke exposure [15] and the 175 genes identified by the linear model.

**Figure 1:**
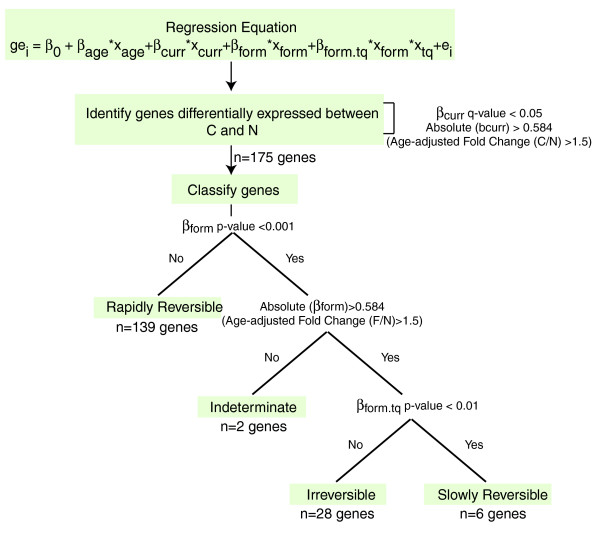
Methodology for gene classification by degree of reversibility upon smoking cessation. For each probeset, the relationship between gene expression in log_2 _scale (ge), age, current smoking status (x_curr_), former smoking status (x_form_), and the interaction between former smoking status and months elapsed since quitting smoking (x_tq_) was examined with the linear regression model. Genes differentially expressed between current (C) and never (N) smokers were categorized based on their behavior in former smokers (F) relative to never smokers as a function of time since smoking cessation. Genes were classified as 'rapidly reversible' if there was not a significant difference between former and never smokers. Genes were classified as 'indeterminate' if there was a significant difference between former and never smokers, but the age-adjusted fold change between former and never smokers was not greater than or equal to 1.5. If the fold change criterion was met, genes were classified as 'slowly reversible' if there was a significant relationship between gene expression and time since quitting smoking or as 'irreversible' if there was not a significant relationship with time.

**Figure 2:**
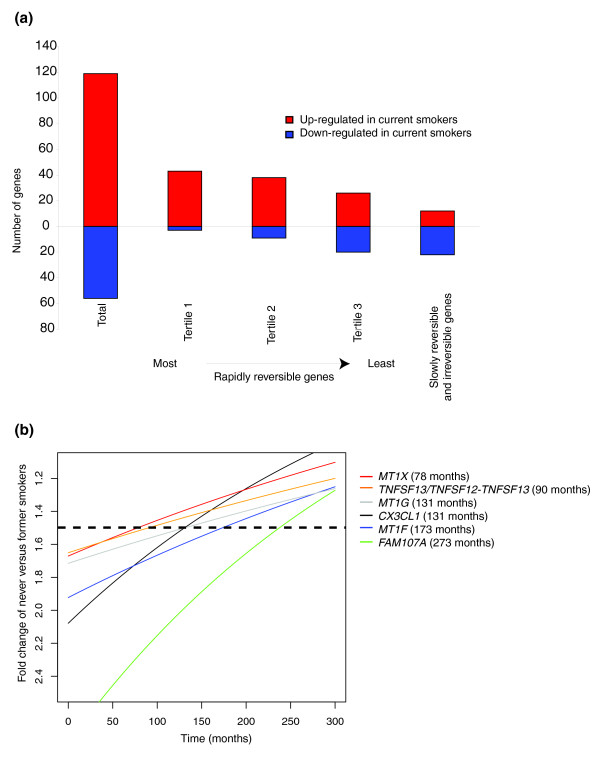
Characteristics of genes classified as irreversible, slowly reversible, or rapidly reversible based on their behavior in former smokers. **(a) **Numbers of genes up-regulated (red) or down-regulated (blue) in current smokers compared to never smokers. The percentage of genes up-regulated in smoking decreases from the most to the least reversible tertile of rapidly reversible genes and is lowest in the slowly reversible and irreversible genes. **(b) **The age-adjusted fold change between never versus former smokers (y-axis) is plotted as a function of time since quitting smoking (x-axis) for the genes classified as slowly reversible. All the slowly reversible genes are down-regulated in smoking. The time point that the fold change equals 1.5 (see dotted line) is defined as the time that the genes become reversible. The time point at which this occurs is greater than 78 months (6.5 years) after smoking cessation for all of the slowly reversible genes.

**Figure 3:**
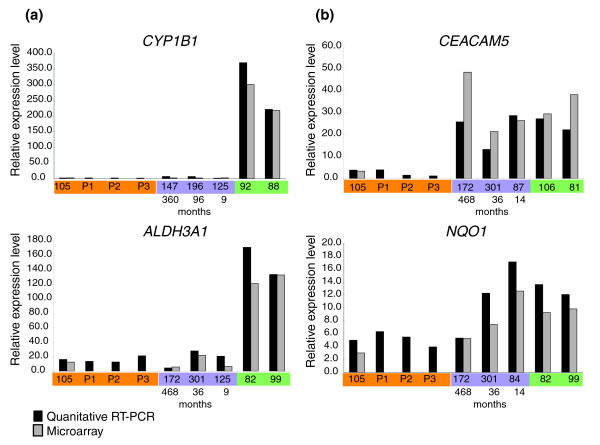
Quantitative real time PCR results for select genes across never, former, and current smokers. For each graph sample identifiers for never (orange), former (purple), and current (green) smokers are listed along the x-axis. The sample identifications P1, P2, and P3 refer to three samples collected prospectively from never smokers that do not have corresponding microarrays. The months since smoking cessation are listed below each former smoker. The relative expression level on the y-axis is the ratio of the expression level of a particular sample versus that of a dummy reference sample. **(a) **Plots of two rapidly reversible genes, *CYP1B1 *and *ALDH3A1*. **(b) **Plots of two irreversible genes, *CEACAM5 *and *NQO1*.

The 175 genes differentially expressed between current and never smokers were classified as irreversible, slowly reversible, or rapidly reversible based on their behavior in former smokers (Figure 1). This yielded 28 irreversible genes, 6 slowly reversible genes, 139 rapidly reversible genes, and 2 indeterminate genes. The 139 rapidly reversible genes were subsequently divided into three equal tertiles based on their percent reversibility (see Materials and methods; Figure 2a). Genes classified as slowly reversible were characterized by the time point at which the age-adjusted fold change between never and former smokers dropped below the threshold of 1.5 (see Materials and methods). The time point is greater than 78 months for all of the genes classified as slowly reversible (Figure 2b). A list of the 175 genes as well as their reversibility classification and percentage is displayed in Additional data file 1. The gene expression level was confirmed by quantitative real time PCR for two irreversible and two rapidly reversible genes (Figure 3).

Interestingly, 65% of the slowly reversible and irreversible genes were down-regulated by smoking, while only 23% of rapidly reversible genes were down-regulated by smoking (Fisher exact test *P *= 7.2 × 10^-6^). Amongst the rapidly reversible genes, those that were down-regulated tended to be the least reversible as determined by percent reversibility (Fisher exact test *P *= 0.0001 comparing the proportion of down-regulated genes in each tertile). Genes down-regulated by smoking, for example, account for only 6.5% of the most reversible tertile of rapidly reversible genes (*n *= 46), but account for 43% of the least reversible tertile (Figure 2a).

**Figure 4:**
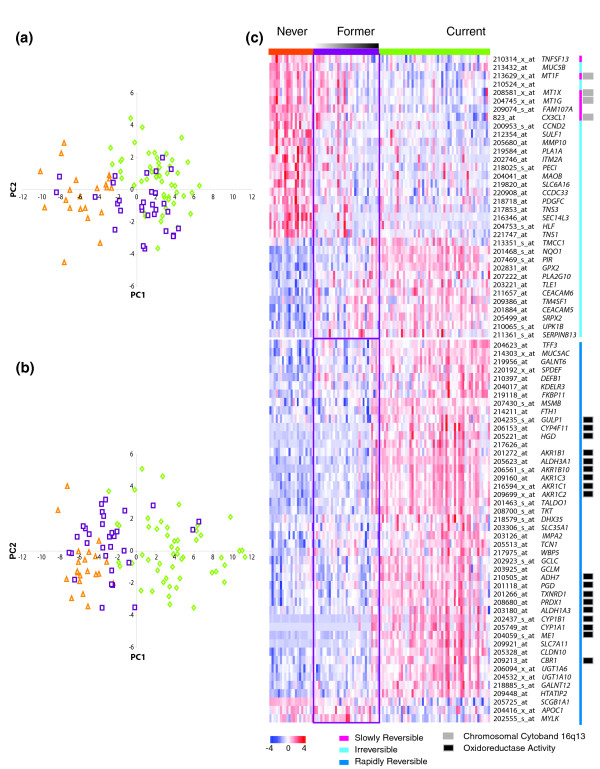
Relationship between samples according to the expression of genes with different reversibility characteristics. PCAs are shown on the left for **(a) **the slowly reversible and irreversible genes (*n *= 34) and **(b) **the most rapidly reversible genes (*n *= 46). **(c) **False-color heatmaps are shown on the right for the slowly reversible and irreversible genes (top) and the most reversible tertile of rapidly reversible genes (bottom). Never, former, and current smokers are colored in orange, purple, and green respectively. The PCA and heatmaps were constructed using gene expression data normalized to a mean of zero and a standard deviation of 1. Never and current smokers are organized according to increasing age and former smokers are ordered by decreasing time since quitting smoking (denoted by the gradient) along the sample axis in the heatmap. Affymetrix identifications and HUGO gene symbols are listed for each gene as well as membership in two over-represented functional categories by EASE analysis.

As expected, a principal component analysis (PCA) using the irreversible and slowly reversible genes shows that former smokers are similar to current smokers (Figure 4a), while a PCA using the most reversible tertile of rapidly reversible genes demonstrates the reverse (Figure 4b). The PCA analyses also demonstrate heterogeneity among former smokers. There are 3 former smokers (time since quit smoking 96, 156, and 300 months) in Figure 4a that cluster with the never smokers and 3 former smokers (time since quit smoking 3, 6, and 14 months) in Figure 4b that cluster with the current smokers, raising the possibility that these individuals may have a different physiological response to tobacco smoke. A heatmap of the gene expression levels of never, former, and current smokers across the slowly reversible and irreversible genes as well as the most reversible tertile of rapidly reversible genes demonstrates the greater proportion of genes down-regulated by smoking among the irreversible and slowly reversible genes (Figure 4c).

EASE [16] was used to identify which Gene Ontology (GO) molecular function categories [17], KEGG pathways [18], GenMAPP pathways [19], and chromosomal cytobands are over-represented (Permutation *P *≤ 0.01) among genes designated as irreversible and slowly reversible or reversible compared to all annotated genes on the Affymetrix U133A microarray (Table 2). The metallothioneins (*MT1G*, *MT1X*, and *MT1F*) and the chemokine *CX3CL1 *are located on Cytoband 16q13, which is over-represented among irreversible and slowly reversible genes (Figure 4a). Although not all metallothioneins in the region of 16q13 were present in the list of 175 genes, all of the probesets on the U133A corresponding to *MT4*, *MT3*, *MT2A*, *MT1E*, *MT1M*, *MT1F*, *MT1G*, *MT1H*, and *MT1X *were down-regulated in current smokers. Genes involved in the metabolism of the carcinogenic components of cigarette smoke, including electron transporter activity and oxidoreductase activity, are over-represented among the rapidly reversible genes. Genes with oxidoreductase activity, such as the aldo-keto reductases, aldehyde dehydrogenases, and the cytochrome p450s, were predominantly present in the most reversible tertile of the rapidly reversible genes (Fisher Exact *P *= 1.3 × 10^-5 ^comparing the proportions of genes in each tertile with oxidoreductase activity; Figure 4c).

**Table 2:** EASE analysis results

System	Category	EASE score	Permutation P value	*R*eversibility group
GO molecular function	Oxidoreductase activity	8.49E-08	1.00E-03	Rapidly reversible genes
GO molecular function	Electron transporter activity	4.60E-06	1.00E-03	Rapidly reversible genes
GenMAPP pathway	*Homo sapiens*pentose phosphate pathway	8.59E-06	1.00E-03	Rapidly reversible genes
GO molecular function	Oxidoreductase activity, acting on the CH-OH group of donors, NAD or NADP as acceptor	5.73E-05	2.00E-03	Rapidly reversible genes
GO molecular function	Oxidoreductase activity, acting on CH-OH group of donors	7.59E-05	2.00E-03	Rapidly reversible genes
KEGG Pathway	Carbohydrate metabolism - *Homo sapiens*	1.71E-04	4.00E-03	Rapidly reversible genes
Chromosomal location	16q13	2.02E-03	1.00E-03	Slowly reversible and irreversible genes

EASE was used to identify GO molecular function categories, KEGG pathways, GenMAPP pathways, and chromosomal locations over-represented (Permutation *P *≤ 0.01) among genes designated as slowly reversible and irreversible or rapidly reversible compared to all annotated genes on the Affymetrix U133A microarray.

### Enrichment of irreversible and reversible genes in bronchial epithelial cell datasets

**Figure 5:**
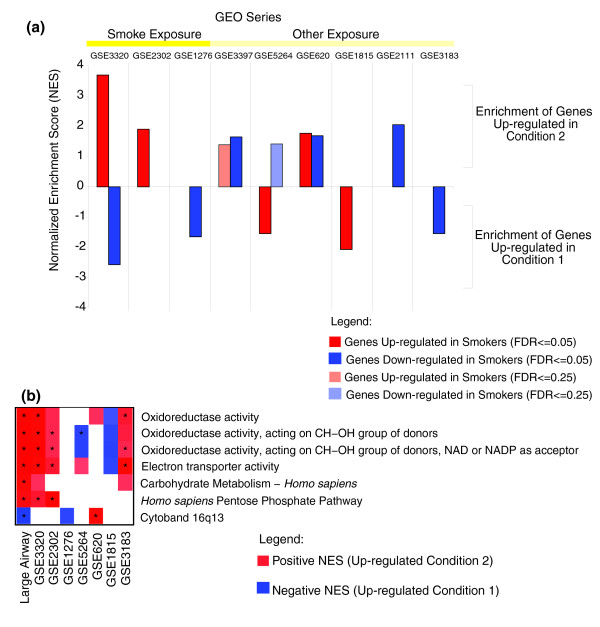
Similarities and differences between our dataset and other bronchial airway datasets. **(a) **GSEA was used to determine if there was a gene expression relationship between other airway datasets (see Table 3 for a description of conditions 1 and 2) and our dataset based on the genes we identified to be regulated by smoking. The normalized enrichment score is plotted for datasets that had a FDR < 0.25. **(b) **Gene lists derived from functional categories and chromosomal locations found to be over-represented by EASE analysis in our dataset were tested for enrichment in our dataset and the other ten datasets using GSEA. A false-color heatmap of the positive (red) and negative (blue) normalized enrichment scores (with a FDR < 0.25) is shown for each category. An asterisk indicates the results passed a stricter FDR < 0.05. The nine datasets and conditions that yielded significant results in either (a) or (b) are indicated in Table 3 by the presence of a single asterisk.

In order to confirm the impact of smoking on airway epithelial cell gene expression and examine the specificity of this response, we compared our findings with ten other previously published human bronchial airway epithelial cell microarray datasets involving a variety of exposures (Additional data file 2). PCAs were performed for each of the 10 datasets across the 175 genes (differentially expressed between never and current smokers) that could be mapped to the microarray platform used in each study using gene symbols (data not shown). Of the 175 genes, 173 had gene symbols, and all of these mapped to the following datasets: GSE5264, GSE3397, GSE3320 GSE3183, GSE2111, and GSE620. One-hundred forty-nine genes mapped to GSE2302 and GSE1276, and 135 genes mapped to datasets GSE1815 and GSE3004. The relationship between the experimental conditions studied in each of the Gene Expression Omnibus (GEO) datasets to our dataset was defined using gene set enrichment analysis (GSEA; Table 3). Significant GSEA results (p value < 0.05 and false discovery rate (FDR) < 0.25) are displayed in Figure 5a. Genes that are perturbed by smoking in the present study are also enriched or differentially expressed (by the signal to noise metric [20]) in the three smoking datasets, corroborating the gene expression changes identified by the linear model. Genes up- and down-regulated by smoking in our dataset were most closely related to (had the highest enrichment scores) genes differentially expressed in dataset GSE3320. GSE3320 was generated using epithelial cells obtained from the small airways (10th to 12th order) at bronchoscopy from both non-smoking and smoking volunteers, and is thus the most closely related to our dataset [21]. Genes up-regulated by smoking in our dataset are also up-regulated in dataset GSE2302. The lack of enrichment in genes down-regulated by smoking in our dataset and genes down-regulated in GSE2302 may reflect differences between the effects of acute and chronic cigarette smoke exposure; our study is likely to capture the gene expression consequences of chronic exposure while bronchial cell cultures in the GSE2302 series were exposed to smoke for only 15 minutes and assayed at 4 and 24 hour time points after the exposure.

**Table 3:** Two group comparisons examined for each of the GEO datasets

Dataset	Condition 1	Condition 2	No. of samples in condition 1	No. of samples in condition 2	Significant dataset
GSE3320	Non smokers	Smokers	5	6	*
GSE2302	Control	Smoke 15 min, 24 hr recovery	9	5	**
GSE2302	Control	Smoke 15 min, 4 and 24 hr recovery	9	9	*
GSE2302	Control	Smoke 15 min, 4 hr recovery	9	4	**
GSE1276	Untreated, 2 and 4 h S9+CSCA/CSCB	8 and 12 h S9+CSCA/CSCB	10	8	*
GSE1276	S9 2, 4, 8 and 12 h	S9+CSCA 2, 4, 8, 12 h	8	8	
GSE1276	S9 2, 4, 8 and 12 h	S9+CSCB 2, 4, 8, 12 h	8	8	
GSE1815	Untreated 8 and 24 h	INF-gamma treated 24 h	9	5	**
GSE1815	Untreated 8 and 24 h	INF-gamma treated 8 and 24 h	9	9	*
GSE2111	Control	Zinc sulfate	4	4	*
GSE2111	Control	Vanadium	4	4	
GSE5264	Days 0 through 8	Days 10 through 28	14	16	*
GSE620	Control	4-PBA 12 and 24 h	5	6	*
GSE620	Control	4-PBA 24 h	5	3	**
GSE3397	Control	RSV 24 h	4	4	
GSE3397	Control	RSV 4 and 24 h	4	8	*
GSE3183	Control	IL13 4, 12, and 24 h	6	9	
GSE3183	Control	IL13 24 h	6	3	
GSE3183	Control + IL13 4 h	IL13 12 and 24 h	9	6	*
GSE3004	Pre-allergen challenge	Post-allergen challenge	5	5	

The GEO series accessions as well as the description and numbers of samples in each of the conditions are listed for each comparison. Datasets where genes differentially expressed between condition 1 and condition 2 demonstrated similarity to genes differentially expressed between current and never smokers in our dataset are indicated by the presence of one or two asterisks. Only comparisons indicated by a single asterisk are shown in Figure 5. S9, rat S9 microsomal fraction; CSC, cigarette smoke condensate; INF-gamma, interferon gamma; 4-PBA, 4-phenylbutyrate; RSV, respiratory syncytial virus; IL13, interleukin 13.

In contrast to the above two datasets, the similarity between the gene expression changes in our dataset and those in GSE1276 was not as strong. GSE1276 used bronchial epithelial cells obtained from cadavers to study the effects of the S9 microsomal fraction from 1254-Aroclor treated rats and cigarette smoke condensate from two different brands of cigarettes at 2, 4, 8, and 12 hour time points [22]. Genes down-regulated by smoking in our dataset were also down-regulated in epithelial cells treated with S9 plus cigarette smoke condensate for 8 and 12 hours compared to earlier time points. The uniqueness of GSE1276 is potentially due to the S9 treatment, which had unexpected broad effects on gene expression that may enhance or suppress the effects of the tobacco smoke condensate [22].

Genes that are perturbed by tobacco smoke exposure in our dataset also show some evidence of differential expression in six out of seven additional bronchial epithelial cell datasets. Genes up-regulated by smoking tended to be genes that are down-regulated by interferon gamma treatment for 24 hours in (GSE1815) [23], suggesting that smoking may have an immunosuppressive effect. Genes up-regulated in smoking also tended to be genes that are down-regulated at later time points during mucociliary differentiation (GSE5264) [24], suggesting that the damage caused by tobacco-smoke induces genes that are expressed more highly in undifferentiated epithelial cells. Genes down-regulated by smoking tended to be genes that are up-regulated in response to zinc sulfate (GSE2111) [25]. These included the metallothionein genes (*MT1X*, *MT1F*, and *MT1G*). Taken together, the above results suggest that the bronchial epithelial cell response to tobacco smoke exposure consists of components that are shared with the response to a variety of other exposures.

### Identifying common biological themes across datasets

In order to build upon the relationships between the datasets described above, we sought to establish additional relationships at the functional or pathway level. Gene lists composed of the genes in each of the over-represented gene categories (Table 2) were used to determine if these gene categories tended to be differentially expressed in the other bronchial cell datasets using GSEA (Figure 5b). This analysis shows that genes in five of the six functional categories that are induced by smoking and rapidly reversible upon smoking cessation also tended to be differentially expressed in two of the three smoking datasets. This further strengthens the notion that a similar bronchial epithelial response to tobacco smoke exposure is being detected in these datasets. Additionally, genes involved in oxidoreductase activity (which we found to be induced by smoking and rapidly reversible upon smoking cessation) are enriched among genes down-regulated during differentiation (GSE5264) or in response to interferon gamma treatment (GSE1815). These genes are also enriched among genes up-regulated in response to 4-phenylbutyrate (4-PBA) (GSE620) or interleukin-13 (GSE3183).

### Biomarker of past exposure

Irreversible gene expression changes in response to tobacco smoke exposure suggest that a gene expression biomarker can be developed that indicates whether an individual has ever been exposed to tobacco smoke. The ability of such a biomarker to accurately classify additional former smoker samples would serve as an important validation of the irreversible gene expression changes we identified. A biomarker of tobacco exposure was constructed using the 28 irreversible genes and a training set of never and former smokers from our primary dataset (*n *= 52). A support vector machine (SVM) classifier was able to classify 100% of the training set samples correctly. The SVM was then first used to predict the tobacco exposure status of the current smokers in our dataset. Not surprisingly, as these samples were used to define the 28 irreversible genes despite having not used these samples to develop the SVM, the SVM correctly predicted 89% of current smokers as having had exposure to cigarette smoke. The 6 current smokers predicted incorrectly had low pack-years (average was 9.5 in contrast to the group average of 34.5). In addition, current and former smokers from a previous study (GSE4115) [26] that did not overlap with the samples used in this study were used as an additional test set. In this dataset, the SVM correctly classified 100% of current smokers and 81% of former smokers. Dividing the former smokers from dataset GSE4115 into 3 groups, former smokers who quit less than 2 years ago (*n *= 12), former smokers who quit greater than or equal to 2 years but less than 10 years ago (*n *= 15), and former smokers who quit greater than or equal to 10 years ago (*n *= 20) yielded similar accuracies (83%, 80%, and 80%, respectively). Finally, the SVM correctly predicted the class of all samples from non-smokers (*n *= 4) and 80% of samples from smokers (*n *= 5) from a recently published dataset (GSE5372). The accuracy of the biomarker in predicting samples from datasets GSE4115 and GSE5372 was significantly better than the accuracies obtained in 1,000 runs that trained the SVM on class-randomized training sets (*P *= 0.01 and *P *= 0.001, respectively; Table 4).

**Table 4:** Biomarker of tobacco smoke exposure constructed using the 28 irreversible genes

	Training set			Test set	GSE4115			GSE5372		
				
	Never	Former	All	Current	Former	Current	All	Non-smokers	Smokers	All
Number Classified Correctly	21	31	52	46	38	38	76	4	4	8
Total Number	21	31	52	52	47	38	85	4	5	9
Accuracy	100.0%	100.0%	100.0%	88.5%	80.9%	100.0%	89.4%	100.0%	80.0%	88.9%
Mean of Random sets			52.5%	59.2%			59.2%			50.1%
*P *value			0	0.102			0.013			0.001

The accuracy of the biomarker is reported for the training set samples, test set samples, samples from dataset GSE4115 that do not overlap with the present study, and samples from GSE5372. The *P *values represent the proportion of 1,000 random training sets that have the same or better accuracy on the tested samples as the actual biomarker.

## Discussion

Using linear models, we have identified genes differentially expressed in airway epithelium between never and current smokers and have characterized expression levels of these genes in former smokers who quit smoking for different periods of time. The majority (79%) of genes differentially expressed between current and never smokers are rapidly reversible upon smoking cessation while the remainders are either slowly reversible or irreversible. Differences between the rapidly reversible and slowly reversible or irreversible genes further suggest that their expression might be regulated through different mechanisms. The rapidly reversible genes have different biological functions than the slowly reversible or irreversible genes, suggesting that they might distinguish between an acute response to tobacco smoke and a more long-lasting response to tobacco smoke induced epithelial cell damage. The gene expression consequences of tobacco smoke exposure we identified are similar to gene expression changes observed in other human bronchial airway gene expression datasets involving tobacco smoke. Commonalities with human bronchial airway datasets involving other exposures suggest that the response to tobacco smoke exposure involves a number of common bronchial airway pathways. The accuracy of a biomarker of tobacco smoke exposure using irreversible genes in additional samples suggests that the irreversibility of these gene expression changes may provide a useful tool for assessing past exposure to tobacco smoke.

Many of the rapidly reversible genes are up-regulated by smoking and are involved in a protective or adaptive response to tobacco exposure and the detoxification of tobacco smoke components. The cytochrome p450s, *CYP1A1 *and *CYP1B1*, for example, are among the rapidly reversible genes and are involved in the oxidation of many compounds, including fatty acids, steroids, and xenobiotics. *CYP1A1 *and *CYP1B1 *have been previously described as being up-regulated in response to smoke [27] and *CYP1B1 *polymorphisms can influence the risk of developing lung cancer among never smokers [28]. Several aldo-keto reductases, like *AKR1B10 *and *AKR1C1*, are also rapidly reversible upon smoking cessation. Aldo-keto reductases are soluble NADPH oxidoreductases that are involved in the activation of polycyclic aromatic hydrocarbons present in tobacco smoke and in the detoxification of highly carcinogenic nicotine-derived nitrosamino-ketone (NNK) compounds [29]. Another class of rapidly reversible genes are the aldehyde dehydrogenases, such as *ALDH3A1*, which are involved in the oxidation of toxic aldehydes produced from oxidative stress and exposure to tobacco smoke [30]. Both the cytochrome p450s and the aldehyde dehydrogenases have been found to be up-regulated in respiratory tissue from rats exposed to smoke [31] and the aldo-keto reductases are up-regulated in normal bronchial epithelium and non-small cell lung tumor tissue from smokers compared with non-smokers [32]. All of the genes listed above as well as most of the differentially expressed genes that are members of the GO molecular function category 'oxidoreductase activity' are among the most highly reversible genes, suggesting that the up-regulation of these genes is driven by the acute exposure to smoke-related toxins and returns to baseline soon after the exposure to these compounds ceases. The induction of these genes in airway epithelial cells after 15 minutes of exposure to tobacco smoke (GSE2302) lends further support to this hypothesis.

In contrast to the rapidly reversible genes, the slowly reversible and irreversible genes reflect a more permanent host-response to tobacco smoke. Interestingly, several of these genes have been associated with the development of cancers of epithelial origin. *CEACAM5*, carcinoembryonic antigen-related cell adhesion molecule 5, is irreversibly up-regulated by smoking and is elevated in the serum of cancer patients with lung adenocarcinoma [33] and colorectal cancer [34]. *SULF1 *(sulfatase 1), a gene irreversibly down-regulated by smoking, influences the sulfation state of residues present on heparin sulfate proteoglycans, which are involved in cell adhesion and mediate growth factor signaling. *SULF1 *was found to be down-regulated in ovarian, breast, pancreatic, renal, and hepatocellular carcinoma cell lines [35] and head and neck squamous carcinomas [36]. *UPK1B*, uroplakin 1B, plays a role in strengthening and stabilizing the apical cell surface through interactions with the cytoskeleton [37]. *UPK1B *is irreversibly down-regulated by smoking and has been shown to be reduced or absent in bladder carcinomas through CpG methylation of the proximal promoter [38,39].

The enrichment of down-regulated genes among the irreversible, slowly reversible, and the least rapidly reversible genes suggests that genetic or epigenetic mechanisms, such as chromosomal loss [7,8] or changes to promoter methylation status [11,12], might account for the relative permanence of these gene expression differences. Given the rather rapid turnover of airway epithelial cells, the persistence of these changes post-smoking cessation may result from a clonal growth advantage to epithelial cells in the airway harboring these changes. Several of the down-regulated slowly reversible genes are present in cytoband 16q13, where a number of metallothioneins are located. Metallothioneins have the ability to bind both essential metals, like copper and iron, as well as toxic metals, such as cadmium and mercury. They also have detoxification and antioxidant properties and may be involved in cell proliferation and differentiation [40]. *MT3 *has been shown to be down-regulated by hypermethylation in non-small cell lung tumors and cell lines [41]. In addition, metallothioneins are thought to regulate some zinc-dependent transcription factors, such as the tumor suppressor *p53*, by donating zinc [42]. Potential loss or methylation of the chromosomal locus containing several metallothionein genes may impair the ability of epithelial cells to protect or to repair cellular injury from future environmental exposures that occur after smoking cessation.

In order to confirm the observed effect of smoking and smoking cessation described above, we compared our dataset with other publicly available human bronchial epithelial cell datasets involving a variety of exposures. Reproducibility of findings using different microarray datasets across similar experimental conditions and cell types has not traditionally been common practice because overlap between differentially expressed gene sets is often surprisingly small [43]. New methodologies for comparing datasets make the task more feasible [44], and provide more powerful methods for determining commonalities between the observed responses of a particular cell type under one or more conditions. The tobacco exposure associated gene expression changes we observed were concordant in three other datasets involving tobacco smoke exposures. The most significant similarity involved the gene expression consequences of tobacco smoke exposure in the small airway epithelium of never and current smokers (GSE3320). This suggests that the field of injury in response to tobacco smoke is similar throughout both the large and small airways. There was also significant similarity between those genes we found to be up-regulated by smoking and the immediate gene expression changes resulting from acute tobacco exposure (GSE2302). This similarity was significant for both rapidly reversible and irreversible/slowly reversible up-regulated genes (data not shown). The lack of similarity among genes down-regulated by smoking in our dataset and GSE2302 may reflect differences between acute and chronic cigarette smoke exposure, and suggests that up- and down-regulated irreversible gene expression may occur through different biological mechanisms. Additional large datasets of acute and chronic tobacco smoke exposure are needed to further explore these hypotheses.

There were also significant similarities between genes up- and down-regulated by smoking and the gene expression differences in additional datasets such as GSE5264 (cells undergoing mucociliary differentiation) and GSE1815 (interferon gamma treated cells). These may provide biological insights about the nature of airway epithelial response to tobacco smoke exposure. The gene expression program that accompanies mucociliary differentiation has led to the hypothesis that cultured 'undifferentiated' epithelial cells may more closely resemble damaged epithelium or neoplastic lesions *in vivo *because many genes associated with normal squamous epithelia, squamous cell carcinomas, or epidermal growth factor receptor signaling are more highly expressed in undifferentiated cells [24]. The similarity between genes up-regulated by smoking in our dataset and genes that are more highly expressed early in mucociliary differentiation together with the similarity between genes down-regulated by smoking in our dataset and genes that are more highly expressed late in mucociliary differentiation might, therefore, reflect the cellular damage induced by smoke exposure. In addition, there was similarity between genes up-regulated by smoking in our dataset and genes down-regulated by treatment with interferon gamma. As interferon gamma plays a role in lung inflammatory responses, these similarities suggest that tobacco smoke exposure may suppress inflammatory responses in the airway. The relationships described above and presented in the results between our dataset and the other datasets are confirmed at a pathway level and suggest that oxidoreductase activity and electron transporter activity are among the important molecular functions of the bronchial epithelium that are regulated in response to a wide range of carcinogenic, inflammatory, and toxic exposures.

As an additional validation of the gene changes observed in response to smoking and smoking cessation, we developed a biomarker of tobacco smoke exposure. Using genes irreversibly altered by cigarette smoke, we were able to classify an independent sample set of former and current smokers (GSE4115) and a sample set of smokers and non-smokers (GSE5372) with high accuracy. Other datasets examining additional inhaled toxins (for example, ozone or fumes from charcoal stoves) are needed to determine if the persistent genomic changes we have identified are tobacco smoke specific. However, our preliminary biomarker results demonstrate the potential for developing a useful epidemiological tool if the gene expression biomarker could be ultimately extended to less invasive sites, such as the buccal and nasal epithelium, as these are tissues that are also directly exposed to tobacco smoke. Biomarkers of exposure are frequently used to improve upon or validate information about tobacco smoke exposure obtained by questionnaire; however, current biomarkers of tobacco exposure (for example, cotinine [45] and NNAL, a metabolite of the tobacco-specific nitrosamine NNK [46,47]) are limited to detecting recent exposure. Development of a biomarker for long-term past exposure using gene expression could have widespread epidemiological utility. We are further interested to determine if there is sufficient similarity in the gene expression differences caused by distant and low-level tobacco smoke exposure such that a biomarker of past exposure could also detect current or past passive smoke exposure.

## Conclusion

We have, for the first time, categorized smoking-related changes in airway gene expression by their degree of reversibility upon smoking cessation, which begins to provide insights into the mechanisms leading to persistent gene expression changes in the airway epithelium exposed to tobacco smoke. Further understanding of these mechanisms may aid in understanding why former smokers remain at risk for developing lung cancer years after quitting smoking and perhaps aid in developing treatments to lower this risk. In addition, a biomarker of past tobacco smoke exposure based on the expression of the genes that do not return to baseline levels after smoking cessation has the potential to provide a useful tool for epidemiological studies.

## Materials and methods

### Patient population

We obtained airway epithelial brushings from never, current, and former smokers undergoing fiberoptic bronchoscopy between April 2003 and January 2006 (*n *= 281 samples, including replicates (*n *= 12)). Subjects with lung cancer or unknown lung cancer status were excluded from the analyses (*n *= 119). Demographics, including age, pack years, and months since quitting smoking, were obtained from each subject. The subjects were recruited from four institutions: Boston University Medical Center, Boston, MA; Boston Veterans Administration, West Roxbury, MA; Lahey Clinic, Burlington, MA; and St James's Hospital, Dublin, Ireland. The Institutional Review Boards of all of the medical centers approved the study and all subjects provide written informed consent. With the exception of nine samples, all samples used in the analyses were included in studies previously published by our group [15,26,48] (Additional data file 4).

### Airway epithelial cell collection

Bronchial airway epithelial cells were obtained from the right mainstem bronchus with an endoscopic cytobrush (Cellebrity Endoscopic Cytobrush, Boston Scientific, Boston, MA, USA). RNA was isolated and its integrity and epithelial cell content was confirmed as described previously [26].

### Microarray data acquisition

We processed, labeled and hybridized 6-8 μg of total RNA to Affymetrix HG-U133A GeneChips containing 22,283 probesets as described previously [15]. We obtained log_2_-normalized probe-level data using the GCRMA algorithm [49] because it maximized the correlation between technical replicates compared to the Microarray Suite 5.0 algorithm and performed equivalently to a similar method, RMA (robust multichip average) [50] (Additional data file 3). All 281 samples (including replicates) collected during the study period were used for sample filtering. A z-score filter was applied to filter out arrays of poor quality. The filter involves computing an average z-score statistic across all probesets for each sample using z-score normalized data so that the mean gene expression value across all samples for each probeset is 0 and the standard deviation is 1 [26]. Samples with high average z-scores were eliminated in addition to the 119 samples with lung cancer or unknown lung cancer status, leaving 104 samples - 21 never smokers without cancer (N), 31 former smokers without cancer (F), and 52 current smokers without cancer (C). The data can be accessed through GEO accession GSE7895.

### Modeling the effect of smoking and smoking cessation

Linear regression models were used to identify genes differentially expressed as a function of tobacco smoke exposure. These genes were further analyzed to describe gene expression changes upon smoking cessation. For each probeset, the relationship between gene expression in log_2 _scale (ge), age, current smoking status (x_curr _= 1 for current smokers and 0 otherwise), former smoking status (x_form _= 1 for former smokers and 0 otherwise), and the interaction between former smoking status and months elapsed since quitting smoke (x_tq_) was examined with the linear regression model:

*ge*_*i *_= *β*_0 _+ *β*_age _* *x*_*age *_+ *β*_curr _* *x*_*curr *_+ *β*_form _* *x*_*form *_+ *β*_form.tg _* *x*_form _* *x*_*tq *_+ *ε*_*i*_

where ε_i _represents the error that we assumed was normally distributed. The equation describes the expression of a probe i for never and current smokers as:

*Never Smoker*: *ge*_*i *_= *β*_0 _+ *β*_age _* *x*_*age *_+ *ε*_*i*_

*Current Smoker*: *ge*_*i *_= *β*_0 _+ *β*_age _* *x*_*age *_+ *β*_curr _* 1 + *ε*_*i*_

Age was included in the model to control for the potentially confounding effects of age and smoking status (Table 1). By difference, the age-adjusted fold change between current and never smokers is 2^β_curr_. The standard least-square method was used to estimate the regression coefficients, and the significance of the regression coefficients was tested using the *t*-test. Goodness of fit of the models was assessed by analysis of residuals.

Probesets differentially expressed between current and never smokers were defined by two requirements. First, a q-value [51] for the regression coefficient β_curr _< 0.05 (which corresponded to *P *< 7.6 × 10^-4^). The q-value is the expected proportion of false positives incurred when calling probesets with this q-value or smaller significant and was used to correct for multiple comparisons. Second, an absolute value of the β_curr _coefficient >0.584, which corresponds to an age-adjusted fold change of expression >1.5. A fold change cutoff was chosen because of the little power provided by our sample size to detect smaller changes using multivariate linear regression models [52]. After the q-value and fold change criteria were applied, probesets with the same gene symbol (according to the June 2006 HG-U133A Affymetrix annotation files), were filtered such that only the probeset with the lowest q-value was retained. All probesets without gene symbol annotation, however, were included.

The behavior of the probesets selected in the first comparison was further analyzed in former smokers. The linear model shown in equation 1 describes the expression of a probe i in former smokers as:

*Former Smoker*: *ge*_*i *_= *β*_0 _+ *β*_age _* *x*_*age *_+ *β*_form _* 1 + *β*_form.tq _* 1 * *x*_*tq *_+ *ε*_*i*_

and allows us to further classify probes based on the pattern of expression in former smokers as a function of time since quitting smoking with respect to never smokers (Figure 1). From equation 4, we see that the expression of a probeset in a former smoker differs from that of a never smoker if the regression coefficient β_form _is significantly different from 0. The difference can be unrelated to time elapsed since quitting if the regression coefficient β_form.tq _is not significantly different from 0, or it can change over time if β_form.tq _is significantly different from 0. In the latter case, when the changes over time are monotone, we can identify the time point at which the fold change was equal to 1.5 (|*β*_*form *_+ *β*_form.tq _* *x*_form _* *x*_*tq*_| = 0.584). This led us to the following definitions. First, a gene was defined as 'rapidly reversible' if the regression coefficient β_form _was not significantly different from 0 (*P *= 0.001). Second, a gene was defined as 'irreversible' if the regression coefficient β_form.tq _was not significantly different from 0 (*P *= 0.01), but the β_form _coefficient was significantly different from 0 (*P *< 0.001) and the absolute β_form _coefficient was >0.584 (corresponding to an age-adjusted fold change between formers and never smokers >1.5). Third, a gene was defined as 'indeterminate' if the regression coefficient β_form.tq _was not significantly different from 0 (*P *= 0.01), but the β_form _coefficient was significantly different from 0 (*P *< 0.001) and the absolute β_form _coefficient ≤0.584. Fourth, a gene was defined as 'slowly reversible' if the regression coefficients β_form _and β_form.tq _were significantly different from 0 (*P *< 0.001, and *P *< 0.01, respectively) and the absolute β_form _coefficient >0.584. The genes were characterized by the time point (tq) where |*β*_*form *_+ *β*_form.tq _* *x*_form _* *x*_*tq *_| = 0.584. This corresponds to the time point where the age-adjusted fold change of never versus former smokers was equal to 1.5 (since all genes classified as slowly reversible were down-regulated by smoking).

In addition, to characterize the range of reversibility among genes designated as rapidly reversible, the percent reversibility for each gene was calculated according to the formula: $1-\frac{2^{\left|\beta_{form}\right|}}{2^{\left|\beta_{curr}\right|}}$. In rare cases where the former smoker versus never smoker fold change was slightly higher than the current versus never smoker fold change, the percentage was set to 100%; and in cases where the former smokers expression levels returned to a slightly lower level than never smokers, the percentage was set at 0%. The reversible genes were divided into tertiles based on this reversibility percentage.

### Relationship of irreversible and reversible genes to other bronchial epithelial cell datasets

NCBI's microarray data repository, GEO [53], was queried for human bronchial epithelial cell samples in August 2006. Processed data were downloaded from GEO for each dataset (ten datasets total) that contained more than three total samples, contained more than two total samples per condition, and that was processed using whole genome arrays (Additional data file 2). The 175 genes differentially expressed between current and never smokers were mapped to the various datasets. PCAs were performed for each dataset across the mapped probesets using z-score normalized data. Graphs of the first versus second principal component were used as guides to decide what groups of samples show differential expression of the genes we identified as being differentially expressed between current and never smokers (data not shown).

The relationship was subsequently defined quantitatively using GSEA [44] (available through the GenePattern software [54]). The samples in each dataset from above were divided into two groups based on the experimental design - control versus the treated samples. If the samples were treated at two different time points, however, the time points were either combined into one treated group or kept separate for different comparisons between the control and the treated group at a particular time point (the PCAs from above were used to guide these decisions; Table 3). For each comparison, the probesets were mapped to gene symbols using GSEA's Affymetrix annotation files; or, in the case of the two non-Affymetrix arrays (datasets GSE2302 and GSE1276), the annotation file human-library.txt [55] was used. The redundant gene symbols were collapsed using a script written in the R Language for Statistical Computing [56] that retained the probesets with the highest absolute signal to noise ratio. This strategy was chosen so that all potentially differentially expressed genes were included in the analyses. The collapsed datasets were evaluated using GSEA to determine if the gene sets listed below were also differentially expressed in the datasets by the signal to noise statistic comparing treatment versus control. The following gene sets were tested: slowly reversible and irreversible genes up-regulated by smoking; slowly reversible and irreversible genes down-regulated by smoking; rapidly reversible genes up-regulated by smoking; rapidly reversible genes down-regulated by smoking; all genes up-regulated by smoking; all genes down-regulated by smoking. Significant enrichment was defined as a *p *value < 0.05 and a FDR < 0.25 derived using 10,000 gene-label permutations.

### Identifying common biological themes across datasets

EASE [16] was used to identify GO molecular function categories, KEGG pathways, GenMAPP pathways, and chromosomal cytobands over-represented among genes designated as slowly reversible and irreversible or reversible compared to all annotated genes on the Affymetrix U133A microarray (Permutation *P *≤ 0.01). GSEA was subsequently performed using gene lists derived from each significant EASE category to identify which of these over-represented categories were enriched in genes up- or down-regulated in each GEO dataset (Table 3). The enrichment of EASE categories observed in our dataset was confirmed using GSEA in which the β_curr _smoking status coefficient (representing the magnitude of the difference between current and never smokers) was used to order the probesets.

### Biomarker for past smoke exposure

A biomarker of past exposure using the irreversible genes (*n *= 28) was trained on the never and former smokers using a SVM classification system with a linear kernel via the R package e1071 [57]. The SVM model was tested on the training set and three different test sets - the current smokers in the present study, current and former smokers that were not included in the present study from dataset GSE4511 previously published by our group, and GSE5372, which included gene expression measurement from large airway epithelial cells in 4 non-smokers and 5 current smokers at different time points (22 samples total) [58]. The biomarker was used to predict the class of the GSE5372 samples taken at the initial time point (*n *= 9). *P *values for the performance of the biomarker were established by randomizing the class labels of the training set, re-running the algorithm 1,000 times, and calculating the proportion of the random runs that produced biomarkers that had the same or better accuracy in the test set samples.

### Quantitative real time PCR

Quantitative RT-PCR analysis was used to confirm the differential expression of two irreversible and two rapidly reversible genes known to play roles in the detoxification of tobacco smoke and pathogenesis of lung cancer. Primer sequences for the four genes (*ALDH3A1*, *CEACAM5*, *CYP1B1*, *and NQO1*) were designed with PRIMER EXPRESS software (Applied Biosystems, Foster City, CA)) (Additional data file 5). Primer sequences of the housekeeping gene *GAPDH *were adopted from Vandesompele *et al*. [59]. RNA samples (1 μg of residual RNA from the samples used in the microarray analysis) were treated with DNAfree (Ambion, Foster City, CA), according to the manufacturer's protocol, to remove contaminating genomic DNA. Total RNA was reverse-transcribed by using random hexamers (Applied Biosystems) and SuperScript II reverse transcriptase (Invitrogen, Carlsbad, CA). The resulting first-strand cDNA was diluted with nuclease-free water (Ambion) to 4 ng/μl. PCR amplification mixtures (25 μl) contained 20 ng template cDNA, 12.5 μl of 2× SYBR Green PCR master mix (Applied Biosystems) and 300 nM forward and reverse primers. Forty cycles of amplification and data acquisition were carried out in an ABI Prism 7700 Sequence Detector (Applied Biosystems). Threshold determinations were automatically performed by Sequence Detection Software (version 1.9.1; Applied Biosystems) for each reaction. All real-time PCR experiments were carried out in triplicate on each sample (mean of the triplicate shown). Four never, 3 former, and 2 current smokers were chosen for each gene based on the amount of RNA available (17 samples total: 6 current, 7 former, and 1 never smoker from this study and 3 additional never smokers collected prospectively).

### Statistical analysis

All statistical analyses and hierarchical clustering were conducted using R statistical software v 2.2.1 and Bioconductor packages [60].

## Abbreviations

FDR, false discovery rate; GEO, Gene Expression Omnibus; GO, gene ontology; GSEA, gene set enrichment analysis; NNK, nicotine-derived nitrosamino-ketone; PCA, principal component analysis; RMA, robust multichip average; SVM, support vector machine; 4-PBA, 4-phenylbutyrate.

## Authors' contributions

AS, MEL, PS and JB conceptualized and designed the study. AS oversaw patient recruitment, sample acquisition and experimental protocols. MEL, PS and JB contributed to the design of the analytic strategy. JB performed the statistical and computational analyses, interpreted the results, and wrote the manuscript. AS, MEL, PS and JSB supervised the analyses and edited the manuscript. GL performed quantitative RT-PCR and was responsible for QRT-PCR data analysis. AS and JSB supported the work.

## Additional data files

The following additional data are available with the online version of this paper. Additional data file 1 lists classifications of genes differentially expressed between current and never smokers according to their behavior in former smokers. For each gene the following information is given: the Affymetrix identification, the HUGO gene symbol, the direction of the change (up- or down-regulated in current smokers with respect to never smokers), the gene classification based on behavior of former smokers, and the percent reversibility. Additional data file 2 provides a Summary of human bronchial epithelial datasets downloaded from GEO. For each dataset the following information is included: GEO series identification, microarray platform, cell type, where the cells were obtained, cell donor information (if applicable), number of samples, experiment type, exposure, experiment description, data preprocessing, and PUBMED identification (if applicable). Additional data file 3 shows that GCRMA and RMA maximize the correlation between replicate samples. Average Pearson correlations between seven pairs of replicate samples where probeset gene expression values were determined using Microarray Suite 5.0 (MAS 5.0), log-transformed data from Microarray Suite 5.0 (Log_2 _MAS 5.0), and RMA. The average, standard deviation, and median of the correlation coefficients are shown. Additional data file 4 gives GEO identifications for never, former, and current smokers. This file explains how the samples used in the present study overlap with previous publications. GEO identifications are provided for each sample for the present study and for the previously published studies (each study used different data preprocessing). GEO identification 1 refers to the study published in [15] (15210990), GEO identification 2 refers to the study published in [27] (17334370), and GEO identification 3 refers to the present study. The study published in [48] (15608264) did not have an accompanying GEO submission. Additional data file 5 lists the quantitative real time PCR primer sequences. Primer sequences for the four candidate genes (*ALDH3A1*, *CEACAM5*, *CYP1B1*, and *NQO1*) designed with PRIMER EXPRESS software (Applied Biosystems), and the primer sequences of the housekeeping gene *GAPDH *adopted from Vandesompele *et al*. [59].

## Supplementary Material

Additional data file 1 For each gene the following information is given: the Affymetrix identification, the HUGO gene symbol, the direction of the change (up- or down-regulated in current smokers with respect to never smokers), the gene classification based on behavior of former smokers, and the percent reversibility. Click here for file

Additional data file 2 For each dataset the following information is included: GEO series identification, microarray platform, cell type, where the cells were obtained, cell donor information (if applicable), number of samples, experiment type, exposure, experiment description, data preprocessing, and PUBMED identification (if applicable). Click here for file

Additional data file 3 Average Pearson correlations between seven pairs of replicate samples where probeset gene expression values were determined using Microarray Suite 5.0 (MAS 5.0), log-transformed data from Microarray Suite 5.0 (Log_2 _MAS 5.0), and RMA. The average, standard deviation, and median of the correlation coefficients are shown. Click here for file

Additional data file 4 This file explains how the samples used in the present study overlap with previous publications. GEO identifications are provided for each sample for the present study and for the previously published studies (each study used different data preprocessing). GEO identification 1 refers to the study published in [15] (15210990), GEO identification 2 refers to the study published in [27] (17334370), and GEO identification 3 refers to the present study. The study published in [48] (15608264) did not have an accompanying GEO submission. Click here for file

Additional data file 5 Primer sequences for the four candidate genes (*ALDH3A1*, *CEACAM5*, *CYP1B1*, and *NQO1*) designed with PRIMER EXPRESS software (Applied Biosystems), and the primer sequences of the housekeeping gene *GAPDH *adopted from Vandesompele *et al*. [59]. Click here for file
